# Unhealthy food availability, prominence and promotion in a representative sample of supermarkets in Flanders (Belgium): a detailed assessment

**DOI:** 10.1186/s13690-023-01175-3

**Published:** 2023-08-22

**Authors:** Stefanie Vandevijvere, Iris Van Dam, Yasemin Inaç, Vincent Smets

**Affiliations:** Department of Epidemiology and Public Health, J. Wytsmanstraat 14, 1050 Brussels, Belgium

**Keywords:** Supermarkets, Food environments, Shelf length

## Abstract

**Introduction:**

The supermarket food environment is a key setting for potential public health interventions. This study assessed food availability, prominence and promotion in a representative sample of supermarkets in Flanders (Belgium).

**Methods:**

A sample of 55 supermarkets across five chains and 16 Flemish municipalities was selected in 2022, about 64% in the most deprived socioeconomic areas. Healthiness indicators related to food availability (ratio of cumulative linear shelf length for healthy versus unhealthy foods), prominence (proportion of unhealthy foods at check-outs and end-of-aisle endcaps), and promotion (food marketing on food packages) were measured.

**Results:**

Overall, the average ratio of healthy/unhealthy foods in supermarkets in Flanders was 0.36, meaning that for every 10m of shelf length of unhealthy foods there was 3.6m of healthy foods. There was a large variation in ratio’s across supermarket chains. Of all foods available, 97.5% were ultra-processed at the check outs, while 72.2% and 58.5% were ultra-processed at the front and back end-of-aisle end-caps, respectively. Confectionery and sweet biscuits were the food categories with on average the highest number of marketing messages on pack per 10m of shelf length.

**Conclusion:**

Supermarket in-store food environments in Flanders were found generally unhealthy, with those located in low income areas having unhealthier in-store food environments than supermarkets located in medium and high income areas. Despite commitments of all large supermarket chains in Flanders to promote and create healthier in-store food environments, our findings indicate that currently consumers are incentivized to buy unhealthy rather than healthy food products.

**Supplementary Information:**

The online version contains supplementary material available at 10.1186/s13690-023-01175-3.

**Table:** 

**Text box 1. Contribution to the literature**
• This is the first study comprehensively assessing retail food environments in Flanders, Belgium.
• Supermarket food environments were generally unhealthy, with large differences between chains.
• Supermarkets located in low income areas were found to have unhealthier in-store food environments.
• Despite retailer commitments, consumers are incentivized to buy unhealthy rather than healthy foods.

## Introduction

Overweight and obesity rates are increasing across Europe [1]. According to the World Health Organisation (WHO), 59% of adults are currently living with overweight or obesity across the European region [2, 3]. In Belgium the prevalence of overweight was 49% and the prevalence of obesity was 16% in 2018, a significant increase since the first health survey in 1997 (when the prevalence of overweight and obesity were 41% and 11%, respectively). In Flanders the prevalence was slightly lower, about 48% and 15%, respectively in 2018 [4].

One of the main drivers of overweight and obesity are unhealthy obesogenic food environments [5]. These environments have been described as “the collective physical, economic, policy and socio-cultural surroundings, opportunities and conditions that influence people’s food and beverage choices and nutritional status” [6]. Internationally, there is growing interest in improving the healthiness of food environments in order to improve population diets. To date, food environment policies have mainly focussed on food reformulation, fiscal policies (e.g. taxation of sugar sweetened beverages), health-related front-of-pack labelling and improving in-school food environments [7]. However, an additional important setting for potential public health interventions are the supermarket instore food environments, defined as the environments that consumers encounter when buying foods, including the cost, quality, and availability of foods [8]. In Europe, as well as in Belgium, supermarkets are the most important retailers when it comes to grocery shopping [9]. The five leading Belgian retailers (Colruyt, Carrefour, Delhaize, Aldi and Lidl) have a market share of 25% for the sales of packaged foods and 9% for the sales of non-alcoholic beverages for their own-brand products according to Euromonitor data 2018 [10]. Finally, the fact that 59% of the market share is in the hands of those five chains shows that a limited number of supermarkets could influence the purchasing behaviour of a significant proportion of the population through (in-store) health promotion interventions [11].

Previous research already evaluated the nutrition-related commitments of the leading Belgian supermarket chains, the healthiness of their own-brand product portfolios and the food products promoted in their circulars [12, 13]. Results from these studies showed that supermarkets made several commitments regarding the reformulation of their own-brand product portfolio, front-of pack nutrition labelling and limiting marketing toward children. Commitments to improve the healthiness of the instore food environments however, such as dedicating a minimum amount of shelf space to healthy products or limiting the placement of unhealthy products in high prominence areas (e.g. front end-of-aisle endcaps and cash registers), were however often lacking from existing pledges [12]﻿.﻿ It has also been shown that median product portfolios of the leading Belgian supermarkets (own-brand products) consisted for 49% of ultra-processed food products, for 71% of foods not-permitted to be marketed to children and for 41% of products with a Nutri-Score D or E [12]. In addition, foods promoted across circulars consisted for 52% of ultraprocessed products on average, with substantial variations across supermarket chains [13].

Nonetheless, to date, no research in Belgium has assessed the in-store food environments of the biggest supermarket chains. Yet, because of their central role within the Belgian grocery landscape, their in-store food environments present a major opportunity to influence the food choices of a noteworthy proportion of the population.

As such this study aims to, for the first time, assess instore food availability, prominence and promotion in a representative sample of supermarkets in Flanders, Belgium.

## Methods

This is a cross sectional study. Ethics approval for this study was obtained from the Human Participants Ethics Committee of the University of Ghent (reference number ONZ-2022-0138). Informed consent and cooperation for the study was gained from the head offices of the five major Belgian retailers: Delhaize, Colruyt, Carrefour, Lidl and Aldi. Store managers were called by telephone beforehand to discuss the time of the visit. The study methods were explained to them before data collection.

### Sampling of municipalities

**Fig. 1:**
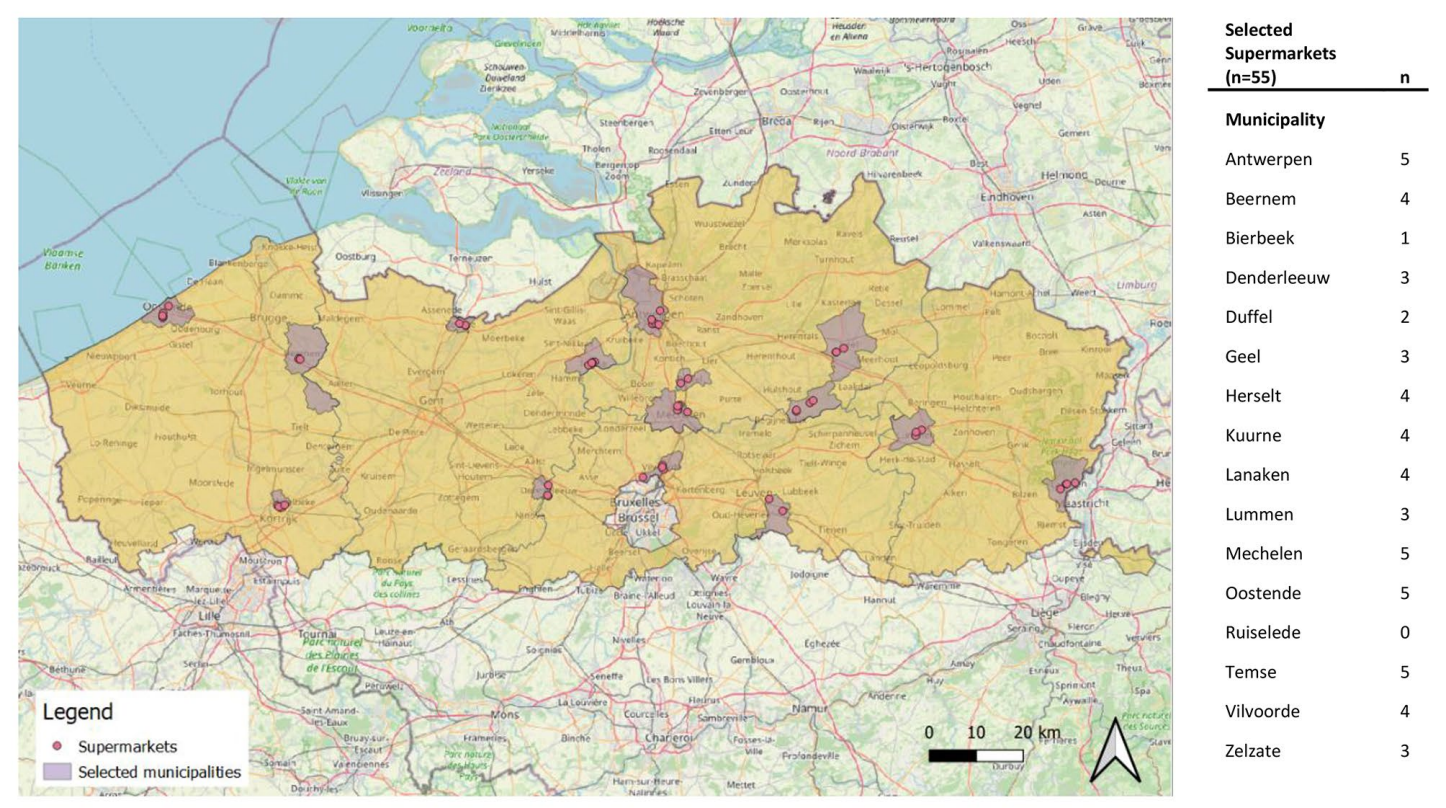
Selection of municipalities and supermarkets in Flanders, 2022

This study forms part of a larger study measuring food environments across diverse settings in Flanders. Based on feasibility, a sample of 16 municipalities out of a total of 300 was selected in Flanders. The following criteria were taken into account when making a representative selection of municipalities: LOGOs (local health care regions [14]), province, population size of the municipality (derived from Statistiek Vlaanderen [15]), demographic data of the municipality (derived from STATBEL [16]), prevalence of overweight and obesity among children (derived from Vlaams Agentschap Zorg en Gezondheid [17]), total number of supermarkets per 1000 inhabitants in the municipality (derived from the Locatus database [18]) and the type of municipality (industrial, agricultural, coastal, urban, rural, etc) (derived from the BELFIUS municipality classification [19]). The final selection of municipalities and some of their socioeconomic indicators are shown in Fig. 1 and Table 1. The level of urbanization of the municipality was derived from Departement Omgeving Vlaanderen [20] and the tertile of median disposable household income was derived from STATBEL [21].

### Sampling of stores

A sample of supermarkets was selected across the five biggest supermarket chains in terms of market share (Colruyt, Carrefour, Delhaize, Lidl, Aldi). One supermarket of each chain (if available) was randomly selected in each selected municipality. and across different urbanization levels and tertiles of median household income for the statistical sectors where the supermarkets were located. In total 55 supermarkets were selected for inclusion and their characteristics are presented in Table 2 and Table 3. The size of the supermarkets (in cm²) was derived from the Locatus database [18].

**Table 1:** Representativeness of the sample of municipalities according to population size, % of elderly, % of young people, yearly net average household income and the total number of supermarkets/1000 inhabitants, 2022

	All municipalities in Flanders (N = 300)	Selected municipalities for the study (N = 16)
Population size (average and SD)	21843 (35731)	38439 (47285)
% Elderly (> 65 years) (average and SD)	21.3 (3.0)	20.7 (3.2)
% Young people (< 15 years) (average and SD)	19.1 (1.9)	19.8 (2.5)
Yearly net average household income (average and SD)	€20717 (2309)	€19926 (1999)
Total number of supermarkets/1000 inhabitants (average and SD)	0.31 (0.12)	0.36 (0.12)

**Table 2:** Total number of supermarkets in Flanders and in the selected municipalities, 2022. Total count and count by chain

Supermarkets Flanders		N		%		
*Total supermarkets in Flanders*		2164		100,0%		
Total supermarkets across 5 largest chains		1037		47,9%		
Delhaize		212		9,8%		
Carrefour		236		10,9%		
Aldi		283		13,1%		
Colruyt		137		6,3%		
Lidl		169		7,8%		
*Supermarkets in selected municipalities*		**N**		**%**		
Total supermarkets		235		100,0%		
Total supermarkets across 5 largest chains		95		40,4%		
Delhaize		17		7,2%		
Carrefour		17		7,2%		
Aldi		25		10,6%		
Colruyt		14		6,0%		
Lidl		22		9,4%		

**Table 3:** The distribution of selected supermarkets across levels of urbanization, income terciles of the statistical sectors where the supermarkets are located and the supermarket chains, 2022

Supermarkets (n = 55)		
	**n**	**%**
Urbanization		
Rural	12	22,2%
Peri-urban	17	31,5%
Urban	25	46,3%
Income tercile		
Low	35	63,6%
Medium	12	21,8%
High	8	14,5%
Supermarket chain		
Delhaize	9	16,4%
Carrefour	9	16,4%
Aldi	14	25,5%
Colruyt	11	20,0%
Lidl	12	21,8%

### Measures

Based on recommendations from the ‘International Network for Food and Obesity/NCDs Research Monitoring and Action Support’ (INFORMAS) [8], the following indicators on the supermarket in-store food environment were measured in this study:

- The ratio of cumulative linear shelf length for healthy versus unhealthy foods
- The cumulative linear shelf length of healthy and unhealthy foods, corrected for supermarket size
- Proportion of foods displayed at the checkouts that are unhealthy
- Proportion of foods displayed at the front end-of-aisle endcaps that are unhealthy

In addition, the extent of marketing on food packages in-store, as well as in different store areas, was assessed.

### Data collection

Data collection was carried out between July and October 2022. For the data collection, five field workers were recruited, one per province in Flanders.

The fieldworkers received a Tablet with the app KoboCollect. All of the data were entered via KoboCollect datasheets, as well as the pictures taken (as explained below). The data were uploaded on a daily basis to the KoboCollect server, after which they were downloaded as excel files and pictures and stored on a local server. The total assessment took between 2 and 3 hours per store, depending on the size of the store and the food products available. A training of two full days was given to the fieldworkers with a practical field work session before the actual start of the field work.

#### Shelf length

For measuring shelf length, six food categories were measured as previous research has shown that these categories are a valid proxy for the overall availability of (un)healthy products in a supermarket [22]. Linear shelf length of 1) fresh fruit and vegetables, 2) frozen fruits and vegetables, 3) soft drinks and energy drinks, 4) crisps and snacks, 5) sweet biscuits, and 6) confectionery was measured by two researchers in centimetres using a MAKA MK201 60M Laster Distance Meter, either along the shelf or along the floor in front of the shelf. The definition of the food categories is given below in Table 4.

**Table 4:** Food categories included in the shelf length measurements in-store

Food group	Description	Classification
Fresh fruit and vegetables	Includes: All fresh fruit and vegetables, packaged fresh fruit and vegetables, fresh herbs Excludes: potatoes, potato products, dried herbs, canned vegetables/fruit, dried fruit	Healthy
Frozen fruits and vegetables	Includes: All frozen fruits and vegetables, as well as mixes without other additives (such as wok vegetables) Excludes: instant and prepared meals, potatoes and potato products	Healthy
Confectionery	Includes: Candy, liquorice, chewing gum (with and without sugar), lollipops and chocolate, candy bars with or without chocolate (e.g. Twix, Mars, M&Ms etc. type). Excludes: dried fruit, sports bars, protein bars, ice creams	Unhealthy
Crisps and snacks	Includes: Chips (all types of chips including nacho and vegetable chips), popcorn, appetiser snacks, crackers Excludes: (salted) nuts, rice cakes with or without chocolate	Unhealthy
Sugar-sweetened beverages	Includes: soft drinks, sports drinks and energy drinks, including light and zero drinks and powders, fruit soft drinks Excludes: fruit juices, waters and flavoured waters, non-alcoholic beer and wine, sugared milk drinks (e.g. chocolate milk)	Unhealthy
Sweet biscuits	Includes: All types of sweet biscuits, cakes and tarts, coffee cakes and pastries Excludes: savoury crackers, rusks and toast, sandwiches	Unhealthy

The number of shelves (of equal measured length) on which the food was displayed was recorded and multiplied by the linear shelf length to obtain the cumulative shelf length for each food category. If shelf length for a particular food category was different across different shelves, the shelf length was measured and recorded for each shelf separately and then summed to produce a total shelf length. For shelving units that did not have a physical shelf (e.g., units with hanging confectionery), rows of hanging products were counted as a single shelf.

Displays that contained multiple rows of different products (e.g., deli meats or dividers between frozen food) were also counted as multiple ‘shelves’. Measurement of islands/freestanding bins was performed by measuring the exposed sides from which customers could pick products, in line with previous studies [23, 24]. For round, freestanding bins, the circumference was measured.

#### Prominence

To measure the prominence of different foods, two different methods were used.

In-store areas (eight areas: 1) check-out end, 1) check-out side, 3) endcaps front, 4) endcaps back, 5) islands, 6) aisles, 7) entrance, and 8) the edges) were categorized into high/medium/low prominence based on the validated Gro Promo tool [25]. The Gro Promo tool measures the locations of the products and assigns them a weighting according to their location. High prominence areas included check-outs (end and side) and endcaps front (endcaps facing the check-outs), medium prominence areas included endcaps back, islands, aisles, and entrance, and the edges of the supermarket were considered low prominence areas [25]. Check-outs included self-check-outs.

Photographs were taken of all food products (and thus not just the selection of food categories taken for the shelf length measures) offered through the checkouts and the end-of aisle endcaps. These were then uploaded in KoboCollect.

#### Marketing on-pack

Marketing was identified for the products photographed in the high prominence locations (check-outs and end-of-aisle endcaps) and for the products within the six food categories for which shelf length was measured. One photo per product of the front of the packaging was taken, taking into account that all extras such as gifts, games, etc. were included in this photo. The brand of the food products was registered.

According to the INFORMAS methodology [8], the following categories were taken into account as marketing:

1. Cartoon character/character owned by the brand (e.g. M&Ms)
2. Licensed character (e.g. Dora the Explorer)
3. Amateur athlete (e.g. a person playing sports)
4. Celebrities (non-sport related)
5. Character associated with film (e.g. Shrek)
6. Famous athlete/team (e.g. famous footballer)
7. Non-sports related/historical events/festivals (e.g. Christmas)
8. For children’ (e.g. image of a child or quote 'ideal for school lunches’)
9. Awards (e.g. Best Food Award 2014, awarded)
10. Sports event (e.g. European Football Championship./World Cup)
11. Other

According to this same INFORMAS methodology [8], the following were considered as premium offers:

1. Game and app downloads
2. Contests
3. Pay 2 take 3 or other
4. 20% extra or other
5. Limited edition
6. Social charity
7. Gift or collectable
8. Price discount
9. Loyalty programs;
10. Other

### Food classification

Foods and non-alcoholic beverages were coded into one of the 17 categories of the WHO Europe nutrient profile model that distinguishes between food products permitted and not permitted to be marketed to children. The model covers all foods and non-alcoholic drinks marketed to or for children aged36 months or older [26]. In addition, all food products were coded according to the extent and purpose of food processing using the NOVA classification [27]. Ultra-processed foods (UPF) are products made mostly or entirely from substances extracted from foods or derived from food constituents with little, if any, intact food. They often contain flavours, colours and other additives that mimic or intensify the sensory qualities of foods or culinary preparations made from foods [27].

### Data analysis

Data were analyzed using SAS statistical software version 9.4 (SAS Institute Inc., 2013). For locations within supermarkets where the number of shelves was not filled in, it was assumed to be 1 shelf. When shelf length was not filled in for some locations and food categories within supermarkets, this was assumed to be zero. When the location at shelf length was not filled in for some food groups, this was included for the total shelf length in the supermarket, but not for the location-specific shelf length. The number of missing data was however very limited (see Annex 1) (< 0.01%).

Descriptive statistics (mean, standard deviation and 95% confidence interval) were used to assess the healthiness of in-store food environments using aforementioned indicators. Analysis of variance tests were conducted to assess differences in indicators between different levels of socioeconomic deprivation (using the tertile of median household income of the areas where supermarkets were located).

## Results

Data were collected for 55 supermarkets in Flanders, of which 31 (56.4%) were in urbanized areas and 33 (60%) in areas with relatively low median household incomes (household incomes within the lowest three deciles) (Table 5).

**Table 5:** Overview of supermarkets included in the study in Flanders, 2022

Brand	N	%	N normal check-outs (N supermarkets)	N self-check-outs (N supermarkets)
Aldi	14	25.5	4 (N = 7), 5 (N = 6), * (N = 1)	0 (N = 13), * (N = 1)
Carrefour	9	16.4	3 (N = 3), 4 (N = 4), 5 (N = 1), 6 (N = 1)	0 (N = 5), 4 (N = 2), 5 (N = 1), 6 (N = 1)
Colruyt	11	20.0	6 (N = 2),7 (N = 1),10 (N = 4),12 (N = 1),13(N = 2), * (N = 1)	0 (N = 10), * (N = 1)
Delhaize	9	16.4	4 (N = 3),5(N = 1),6(N = 4),7(N = 1)	0(N = 5),6(N = 1),8(N = 3)
Lidl	12	21.8	3 (N = 1),5(N = 5),6(N = 3),7(N = 1),10(N = 1), * (N = 1)	0 (N = 11), *(N = 1)

*For 3 supermarkets, the number of check-outs was not entered as it was forgotten by the fieldworkers

The ratios of total cumulative linear shelf length for healthy (fresh and frozen fruit and vegetables) versus unhealthy (soft drinks, confectionery, salty and sweet snacks) foods, as well as the total cumulative linear shelf length for healthy and unhealthy foods, corrected for size of the supermarket, are shown by supermarket chain in Table 6 below.

**Table 6:** Ratio of cumulative linear shelf length for healthy and unhealthy foods for different supermarket chains in Flanders, 2022

		ratio healthy/unhealthy			cm / area in m² for healthy foods			cm / area in m² for unhealthy foods		
Supermarket	N	mean	L 95%CI	U 95% CI	mean	L 95%CI	U 95% CI	mean	L 95%CI	U 95% CI
Aldi	14	0.45	0.38	0.52	8.4	7.4	9.3	19.5	16.3	22.7
Carrefour	9	0.25	0.22	0.28	10.1	8.6	11.6	41.0	35.0	46.9
Colruyt	11	0.39	0.34	0.44	11.6	9.7	13.4	29.9	26.0	33.8
Delhaize	9	0.27	0.24	0.31	9.2	7.2	11.2	34.4	26.1	42.7
Lidl	12	0.39	0.32	0.45	9.6	8.5	10.8	25.3	23.2	27.4
Total	**55**	**0.36**	**0.33**	**0.39**	**9.7**	**9.1**	**10.3**	**28.8**	**26.1**	**31.5**
Total-low SES*	33	0.35	0.31	0.38	9.5	8.9	10.2	29.8	26.2	33.4
Total-medium SES*	12	0.37	0.31	0.43	10.9	8.8	13.0	30.7	34.5	36.8
Total-high SES*	8	0.43	0.30	0.56	8.9	6.8	11.0	22.3	15.8	28.8

* SES - socioeconomic status

Overall, the average ratio of healthy/unhealthy foods in supermarkets in Flanders is 0.36 meaning that for every 10m of shelf length of unhealthy foods there is 3.6m of healthy foods (Table 6). There is large and significant variation in ratio’s across supermarket chains. The discounter Aldi is on average having the highest ratio of healthy versus unhealthy food linear shelf length while Carrefour is having the lowest average ratio.

Corrected for size of the supermarket, Carrefour and Colruyt offer the highest total cumulative linear shelf length for fresh and frozen fruits and vegetables, while this is the lowest for the discounter Aldi, while similarly for unhealthy foods, Carrefour is offering the highest total cumulative linear shelf length and Aldi the lowest (Table 6).

It can also be observed that supermarkets located in more deprived areas have a lower ratio than those located in less deprived areas; for example the shelf length ratio healthy/unhealthy foods is 0.35 on average for supermarkets in areas with the lowest median household incomes while it is 0.43 for supermarkets in areas with the highest median household income (Table 6).

The ratios for total cumulative linear shelf length for healthy (fresh and frozen fruit and vegetables) versus unhealthy (soft drinks, confectionery, salty and sweet snacks) foods, as well as the total cumulative linear shelf length for healthy and unhealthy foods are shown by location in the supermarket and supermarket chain in Table 7 below.

**Table 7:** Ratio of cumulative linear shelf length for healthy and unhealthy foods for different supermarket chains in Flanders by location in the supermarket, 2022

Prominence location	supermarket	mean	L 95%CI	U 95%CI
high	Aldi	0.02	-0.01	0.05
	Carrefour	0.02	-0.02	0.05
	Colruyt	0.13	0.04	0.23
	Delhaize	0.02	-0.03	0.08
	Lidl	0.04	0.00	0.08
	**Total**	**0.05**	**0.02**	**0.07**
medium	Aldi	0.41	0.35	0.47
	Carrefour	0.23	0.19	0.28
	Colruyt	0.16	0.12	0.20
	Delhaize	0.23	0.17	0.29
	Lidl	0.47	0.38	0.57
	**Total**	**0.32**	**0.27**	**0.36**
low	Aldi	3.06	-0.09	6.22
	Carrefour	0.79	0.10	1.49
	Colruyt	3.59	1.41	5.77
	Delhaize	8.85	-1.15	18.85
	Lidl	0.57	0.34	0.79
	**Total**	**3.20**	**1.44**	**4.96**

From Table 7 it can be observed that the shelf length ratio healthy/unhealthy foods is lowest in the high prominence areas such as check-outs and front end-of aisle endcaps while it is highest in the low prominence areas in the supermarket (e.g. along the edge). The differences are substantial. There is also substantial variation across supermarket chains with Colruyt having the highest ratio in the high prominence areas, probably due to the fact that they have a commitment not to have junk food at the check-outs, while Delhaize has the highest ratio in the low prominence areas (Table 7).

When we look more closely at the foods available in the high prominence locations in the supermarkets (front end-of-aisle endcaps and check-outs), we can see that on average 81% of products available are foods and non-alcoholic beverages at the check-outs, while 44% and 52% are foods and beverages for the end-caps front and back, respectively. Of all foods available, 97.5% are ultraprocessed at the checkouts, while 72.2% and 58.5% are ultra-processed at the end-caps front and back, respectively (Table 8). At the check-outs, the most commonly available foods are chocolate and sugar confectionery (71.3%), while at the endcaps front and back the most commonly available foods are chocolate and confectionary (9.4%) and sugary drinks (7.2%) respectively (Table 8).

**Table 8:** Overview of types of foods and brands available at the high prominent locations across supermarkets in Flanders, 2022

Location	% of products that are food	% of products that are alcohol	% of foods that are ultra-processed	% of foods (excl alcohol) by category according to the WHO Europe nutrient profile classification	% of foods (excl alcohol) by brand
Endcap front*	4347 (43.9%)	1086 (10.7%)	3137 (30.5%) 72.2% of total foods	Chocolate and sugar confectionery 9.44 Sugar sweetened beverages6.77 Cakes, sweet biscuits and pastries2.81 Processed meat, poultry, fish, meat replacements2.08 Energy drinks2.04 Savoury snacks (including salted nuts)1.75 Waters, flavoured waters, coffee, tea1.60 Processed fruit, vegetables and legumes1.35 Sauces, dips and dressings1.29 Milk drinks1.10	Verstegen9.06 Carrefour4.77 Ranobo 2.86 Coca-cola2.76 Boni 2.62 Ducros 2.24 Monster 2.21 Look-O-Look2.16 Dolce gusto1.77 Rabeko zero1.72
Endcap back**	4662 (52.1%)	746 (8.3%)	2725 (30.2%) 58.5% of total foods	Sugar sweetened beverages7.22 Chocolate and sugar confectionery 5.92 Waters, flavoured waters, coffee, tea4.10 Processed meat, poultry, fish, meat replacements3.60 Cakes, sweet biscuits and pastries2.78 ND2.69 Savoury snacks (including salted nuts)2.53 Sauces, dips and dressings2.14 Cheeses2.11 Processed fruit, vegetables and legumes1.80	Verstegen4.90 Ducros 3.45 Damhert3.34 Delhaize 3.31 Carrefour3.12 Boni 2.24 Nutribel 2.24 Brets 2.02 Lipton 1.83 Nuts, fruits ´n more 1.61
Check-outs***	5742 (80.5%)	113 (1.6%)	5600 (78.2%) 97.5% of total foods	Chocolate and sugar confectionery 71.33 Cakes, sweet biscuits and pastries1.87 Processed fruit, vegetables and legumes1.54 Savoury snacks (including salted nuts)1.22 Processed meat, poultry, fish, meat replacements0.70 Sugar sweetened beverages0.63 Energy drinks0.54 Unsalted nuts0.21 Ready-made & convenience foods and composite dishes 0.15 Waters, flavoured waters, coffee, tea0.13	Mentos 20.78 Stimorol 8.27 Ricola 7.75 Frisk 6.64 Jet gum 5.82 Haribo 4.30 Kinder 3.87 Tic-tac 2.75 Freedent2.34 Fresh life1.84

*146 of 10279 products were not analyzed due to blurry picture of because of wrong location

**72 of 9021 products were not analyzed due to blurry picture of because of wrong location

***26 of 7160 products were not analyzed due to blurry picture of because of wrong location

When we look at instances of marketing at those high prominence locations (excluding food packages), we observed 26 promotional characters and 97 premium offers for end-caps front, 36 promotional characters and 202 premium offers across the 55 end-caps back and 17 promotional characters and 104 premium offers across all check-outs across the 55 supermarkets investigated (data not shown).

The number of on-pack marketing ads found across the 55 supermarkets is shown below in Table 9. As it concerns marketing on packaging (responsibility of the companies themselves), no breakdown by supermarket is given. Confectionery and sweet biscuits were the food categories with on average the highest number of on pack ads per 10m of shelf length (Table 9). For frozen fruit and vegetables no on-pack marketing was found whilst for fresh fruits and vegetables 54 promotional characters and 1 premium offer were identified (data not shown).For all food groups a large share of the marketing could be attributed to a few brands (Table 9).

**Table 9:** Overview of on-pack marketing found across supermarkets in Flanders, 2022

*Food category*	*Total ads*	*Average number of ads (SD)*	*Average ads/10m shelf length (SD)*	*Top 3 brands*	*% promotional characters*	*% premium offers*
Sugar-sweetened beverages	395	7.2 ± 6.5	0.72 ± 0.57	Twist and drink (25.3%) Oasis (22.0%) Capri-sun (13.9%)	95.4%	25.3%
Confectionery	3190	58.0 ± 32.1	5.8 ± 2.6	Haribo (25.0%) Look-O-Look (12.6%) Lutti (12.5%)	98.6%	9.1%
Crisps and snacks	213	3.9 ± 4.8	0.59 ± 0.56	Croky (47.4%) Lorenz (17.4%) Carrefour (6.1%) Snack day (6.1%)	98.1%	22.5%
Sweet biscuits	1165	21.2 ± 11.7	1.93 ± 0.94	Lu (21.0%) Lotus (12.4%) Sondey (10.8%)	98.5%	3.1%

## Discussion

Supermarket in-store food environments in Flanders are generally unhealthy and do not nudge consumers toward healthier food choices. The measured indicators on availability, prominence and promotion all show a predominance of unhealthy foods, with supermarkets that are located in low-income areas scoring the worst.

Whilst previous research showed that Belgian supermarkets made some nutrition-related commitments to create healthier food environments [12], these commitments mainly relate to reformulation, such as reducing nutrients of concern such as sodium, saturated fat, trans fat, added sugar and energy content; implementing the Nutri-Score on their own-brand food products and committing to the Belgian Pledge to reduce marketing towards children. Nonetheless, commitments regarding product accessibility, such as to dedicate a minimum amount of floor space to healthy products or limit the placement of unhealthy products at high-traffic areas, were mostly lacking [12]. Colruyt however has a commitment not to place unhealthy foods at the check-outs [28] and, while their definition might differ from that used in the study, we indeed observed a very low percentage of ultraprocessed foods at the check-outs for this chain compared to the other chains.

The differences between supermarket chains are stark. The discounters Aldi and Lidl had, together with Colruyt, the best ratio of healthy/unhealthy cumulative shelf length. This is partially in line with a study conducted in Australia, which reported that Aldi had the least amount of space devoted to unhealthy food, in particular at end-of-aisle and checkout displays, in regards to independent and more high-end supermarkets [29]. However, these findings are in contrast with a study from the UK that found that people who bought groceries in discounters bought significantly lower percentages of energy from fruit and vegetables and higher percentages of energy from unhealthy foods [30]. The latter study however did not assess the in-store food environment of supermarkets in the UK and therefore cannot be directly compared with our findings.

That our study found that supermarkets in low income areas have unhealthier in-store food environments might be due to the supply and demand dynamics. Families with a lower socioeconomic position, according to income level or education, may have less nutritional knowledge and material resources (e.g. food budgets and facilities to prepare food) [31, 32] resulting in more unhealthy diets [33]. However, a study which assessed the perceptions of participants with low socioeconomic position towards supermarket nudging (i.e. making healthier choices easier or more intuitive in a retail environment by, for example, placing healthy food items at eye level), found generally positive results regarding the perception of participants towards nudges (34). Unhealthy, ultra-processed foods in Belgium have been found to be less expensive than healthy, unprocessed foods [35, 36]. It is therefore likely that supermarkets located in low income areas change their product promotions and prominence based on their clients preferences.

Marketing aimed at children for unhealthy food categories, such as confectionary and sweet biscuits, is still very common despite the long established scientific findings about its detrimental effects on children’s nutrition knowledge, preference, purchasing behaviour and diet-related health [37]. More than half of the food products in high prominence locations (front end-of-aisle endcaps and checkouts) were ultraprocessed, effectively nudging consumers to buy more of these products [38].

Even though this is a logical action from the supermarkets’ commercial perspective, from a public health point of view this will lead to more health issues of an already more unhealthy and vulnerable part of the population [39].These results underscore the importance of continued and repeated measurements of supermarkets in-store food environments to evaluate the implementation of their commitments.

There are a range of evidence-based actions that food retailers can take to improve the healthiness of retail food environments, categorized broadly into four categories [40]: (1) ‘Corporate strategy’, including actions related to overarching company strategies and goals related to nutrition and health; actively supporting relevant public health-related government interventions; and avoiding lobbying against public health regulations to address unhealthy diets, (2) ‘Product development and labelling’, including actions related to: reformulation of existing own-brand products; introduction of new healthier own brand products; implementation of easy-to-understand food labelling on own-brand products; (3) ‘Product availability and placement’, including actions related to product availability, allocation of shelf space, placement of products in prominent areas, (4) ‘Promotional activities’, including actions related to pricing strategies, promotions in catalogues/circulars, in‑store / online signage, images or branding that appeal to children, loyalty rewards [40]. In most cases, however, mandatory government regulation is likely to be needed to remove commercial barriers (e.g. such as restrictions on price promotions for unhealthy foods) [41].

Strengths of this study include the representative sample of supermarkets included and the diversity of indicators measured covering food availability, prominence and promotion. When using the indicators in future monitoring, it is important to consider the frequency and period of measurements (seasonality, frequency of changes of products within stores). It needs to be acknowledged that the types of foods in some store areas (endcaps) may change more quickly than in others (e.g., check-outs). In addition, the shelf length ratio does not include all foods in-store (e.g., non-food products and products that are not considered either healthy or unhealthy are excluded). Even for the healthy and unhealthy food categories, some indicator food categories were included rather than measuring all foods in order to improve feasibility as the measures of shelf length are the most burdensome to include in the study. Some food products are not placed on a physical shelf (e.g., hanging confectionary or fruit in freestanding bins) and methods were slightly adapted to be able to measure shelf length for those products.

## Conclusion

Supermarket in-store food environments were found to be generally unhealthy, with large differences between supermarket chains and the area they are located. Supermarkets located in low income areas were found to have unhealthier in-store food environments than supermarkets located in medium and high income areas. The cumulative shelf length of healthy to unhealthy food products was under 0.5 for all supermarkets and high prominence areas such as checkouts and front end-of-aisle endcaps predominantly contained unhealthy and ultra-processed foods. Marketing aimed at children was very common for unhealthy food categories such as confectionary and sweet biscuits. Despite commitments of all large supermarket chains in Flanders to promote and create healthier in-store food environments, our findings indicate that currently consumers are incentivized to buy unhealthy rather than healthy food products.

### Electronic Supplementary Material

Below is the link to the electronic supplementary material.

Supplementary Material 1

## Data Availability

The data can be accessed upon reasonable request to the authors

## List of abbreviations

INFORMAS: International Network for Food and Obesity/NCDs Research Monitoring and Action Support
NCDs: noncommunicable diseases
WHO: World Health Organization
