# Supplementary material for: Unhealthy food availability, prominence and promotion in a representative sample of supermarkets in Flanders (Belgium): a detailed assessment
Source: Arch Public Health. 2023 Aug 22;81:154. doi: 10.1186/s13690-023-01175-3 (PMC10463948; doi:10.1186/s13690-023-01175-3)
Supplement: Supplementary file 1 — Supplementary Material 1 [file 13690_2023_1175_MOESM1_ESM.docx]

**Appendix**

Appendix 1 Overview of missing data for the shelf length measurements within supermarkets in Flanders, 2022

| **Food group** | **Number of shelves *** | **Shelf length** | **Location** |
| --- | --- | --- | --- |
| Sugar sweetened beverages | 5/950 | / | / |
| Fresh fruit and vegetables | 1/743 | 1/743 | 1/743 |
| Frozen fruit and vegetables | 1/204 | 3/204 | / |
| Confectionery | 2/942 | 2/942 | / |
| Crisps and snacks | 1/524 | / | 1/524 |
| Sweet biscuits | 3/1041 | 2/1041 | 1/1041 |

*Missing number of shelves was adapted to shelf = 1

Appendix 2 Overview of types of foods and brands available at the high prominent locations across supermarkets in Flanders by SES, 2022

| Supermarket locations | SES | % ultra-processed foods | % alcohol | Top 10 food groups (excl alcohol) % |
| --- | --- | --- | --- | --- |
| Endcaps front | Low (N=6475 products) | 30.1% | 9.9% | 1 9.02  4d 6.42  2 3.38  14 2.37  4c 1.87  17 1.77  16 1.32  8 1.26  4b 1.20  4e 1.07 |
|  | Medium  (2213 products) | 27.8% | 8.9% | 1 8.91  4d 7.22  3a 2.50  4e 2.23  16 1.96  2 1.96  15 1.60  4b 1.47  3b 1.38  9 1.34 |
|  | High (1231 products) | 38.8% | 19.2% | 1 11.53  4d 8.01  4c 5.36  3a 4.72  14 4.00  13 3.92  4e 3.36  2 1.52  15 1.20  3b 1.20 |
| Check-outs | Low (4075 products) | 81.8% | 1.3% | 1 75.86  2 2.22  16 1.08  3a 1.05  4d 0.61 |
|  | Medium (1454 products) | 70.9% | 2.3% | 1 61.05  NA 24.30  A 2.33  2 2.12  3a 1.98 |
|  | High (N=1173 products) | 87.4% | 1.9% | 1 82.45  16 2.21  14 1.11  4d 0.77  17 0.26 |

Appendix 3 Overview of types of foods and brands available at the high prominent locations across supermarkets in Flanders by supermarket brand, 2022

| Supermarket locations | Supermarket chain | % food | % ultra-processed food | % alcohol | Top 5 food groups (excl alcohol) % | Top 5 brands |
| --- | --- | --- | --- | --- | --- | --- |
| Endcaps front | Aldi (N=654) | 46.5% | 30.7% | 27.7% | 1 10.55  4d 7.45  2 2.67  3a 2.25  3b 2.11 | Trader joe´s 7.11  Leffe 4.74  Schweppes 3.56  Château 3.16  Haribo 3.16 |
|  | Carrefour (N=3540) | 48.8% | 26.4% | 9.3% | 4d 6.97  1 4.58  2 3.65  16 2.11  17 2.00 | Verstegen 22.94  Carrefour 12.09  Ducros 5.67  Rabeko zero 3.32  Dolce gusto 2.07 |
|  | Colruyt (N=2130) | 35.7% | 26.2% | 3.8% | 1 14.33  14 1.99  4d 1.85  2 1.76  5 1.71 | Boni 22.30  Milka 3.05  Graindor 2.82  Magnum 2.82  Sodastream 2.82 |
|  | Delhaize (N=3126) | 46.1% | 33.9% | 13.3% | 1 11.54  4d 7.64  4c 4.15  14 2.73  4e 2.22 | Ranobo 7.10  Monster 5.24  Coca-cola 4.88  Delhaize 3.64  Nalu 3.28 |
|  | Lidl (N=683) | 68.5% | 49.8% | 11.7% | 4d 16.45  1 8.37  13 8.23  2 8.08  3a 7.65 | Look-O-Look 10.44  Delacre 9.95  Schweppes 6.31  Duyvis 4.37  Lipton 3.64 |
| Check-outs | Aldi (N=1425) | 84.9% | 82.6% | 0.6% | 1 79.19  14 1.82  2 0.56  16 0.28  17 0.28 | Mentos 20.97  Haribo 17.08  Fresh life 8.89  Ricola 8.02  Stimorol 6.99 |
|  | Carrefour (N=1579) | 90.3% | 88.0% | 0.7% | 1 86.02  3a 0.51  4c 0.38  4d 0.38  16 0.25 | Mentos 22.84  Frisk 10.52  Stimorol 10.52  Ricola 9.80  Tic-tac 4.47 |
|  | Colruyt (N=620) | 1.1% | 1.0% | 0.3% | / | / |
|  | Delhaize (N=1422) | 90.9% | 88.6% | 1.9% | 1 81.16  3a 2.02  4d 1.88  2 1.61  4c 0.98 | Mentos 19.75  Stimorol 13.06  Ricola 10.23  Frisk 7.47  Tic-tac 3.30 |
|  | Lidl (N=2088) | 86.5% | 84.0% | 3.1% | 1 69.55  16 4.74  2 4.74  3a 2.20  4c 0.91 | Mentos 19.88  Jet gum 17.68  Fruit & go 5.07  Delacre 4.79  Sweet corner 4.56 |
